# Supplementary material for: Multilineage Dysplasia as Assessed by Immunophenotype in Acute Myeloid Leukemia: A Prognostic Tool in a Genetically Undefined Category
Source: Cancers (Basel). 2020 Oct 30;12(11):3196. doi: 10.3390/cancers12113196 (PMC7693580; doi:10.3390/cancers12113196)
Supplement: Supplementary file 1 [file cancers-12-03196-s001.pdf]

cancers

MDPI

Supplemental data

Multilineage Dysplasia as Assessed by Immunophenotype in Acute Myeloid Leukemia: A Prognostic Tool in a Genetically Undefined Category

Francesco Mannelli, Sara Bencini, Matteo Piccini, Giacomo Gianfaldoni, Maria Ida Bonetti, Benedetta Peruzzi, Roberto Caporale, Barbara Scappini, Fabiana Pancani, Vanessa Ponziani, Leonardo Signori, Michela Zizza, Francesco Annunziato and Alberto Bosi

Table of Contents

A. Supplemental Materials and Methods

S1. Treatment details

S2. Assessment of multi-lineage dysplasia by multiparameter flow cytometry

B. Supplemental Figures and Legends

S1. Outcome according to the induction regimen, either including standard dose cytarabine (SDAC) or high dose cytarabine (HDAC)

S2. Distribution of phenotypic scores in single cell lineage (neutrophil or erythroid) according to dysplasia by morphology.

S3. Distribution of IPS values according to genotype for NPM1, FLT3 and CEBPA.

S4–S6. Outcome according to immunophenotypic score (IPS) in WHO-defined subsets

S7–S11. Outcome according to immunophenotypic score (IPS) in genetically defined subsets

S12–S13. Outcome according to IPS in triple-negative AML with censoring at allogeneic transplant

S14–S15. Effect of IPS on outcome in triple-negative AML as depicted by Forest plot

S16. Outcome according multi-lineage dysplasia as assessed by morphology in triple-negative AML.

S17–S19. Effect of treatment-related covariates (induction and allogeneic transplant) on outcome in IPS-related groups as depicted by Forest plot

C. Supplemental Tables

S1. Characteristics of patients according to induction regimen (SDAC vs HDAC)

S2. Characteristics of patients according to immunophenotypic score (IPS)

D. References of methods' section

Supplemental Materials and Methods

S1. Treatment Protocols

Protocol-1: since April 2004 to March 2007, patients received induction according to standard-dose cytarabine (SDAC) based course, namely "3+7" (Cytarabine 100 mg/sqm bid on days 1-7; Idarubicin 12 mg/sqm on days 1-3). From 2006 on, etoposide 100 mg/sqm on days 1-5 was added (ICE course). High-dose Cytarabine (3000 mg/sqm bid days 1, 3, 5) was used as first consolidation in patients aged < 61 years attaining complete remission (CR) after ICE. Patients with persistent disease (*i.e.* > 5% BM blasts at hematopoietic recovery) after first course received a salvage regimen (FLA-Ida). In an intention-to-treat approach, patients aged < 55 years with high-risk karyotype, FLT3-ITD or adverse clinical features (secondary AML, CR after second course, hyperleukocytosis) were assigned to undergo allogeneic stem cell transplantation (SCT) from matched related or unrelated donor. Patients with intermediate cytogenetic risk in the absence of FLT3-ITD and adverse clinical features were allocated to allogeneic SCT if a related donor was available. Autologous SCT was offered to patients aged < 61 y with low-risk cytogenetics, intermediate-risk cytogenetics without sibling donor and high-risk disease not eligible to allogeneic SCT. Peripheral blood (PB) stem cells for autologous SCT were collected after a DIA course (Cytarabine 500 mg/sqm bid on days 1-6; Daunorubicin 50 mg/sqm on days 4-6). Patients who failed mobilization received two additional courses with high dose cytarabine.

Protocol-2: since April 2007 to April 2014, patients were treated according to Northern Italy Leukemia Group (NILG) AML 02-06 protocol. Until March 2012, patients were recruited within the NILG AML 02/06 trial [(ClinicalTrials.gov Identifier: NCT00495287][1]. From April 2012, after closure of NILG AML 02/06 trial, patients were treated according to the standard arm provided by the protocol. The protocol provided a randomization at induction between a standard ICE induction *versus* an experimental intensified one. Patients aged > 65 y were treated according to standard arm. Upon CR achievement, patients received standard doses cytarabine consolidation and were divided into standard and high risk cases (SR, HR): SR: favorable or intermediate risk cytogenetics (according to SWOG criteria) without any adverse clinical factor (secondary AML, FLT3-ITD, CR after cycle 2, persistence of pre-existing cytogenetic abnormality despite morphological CR; total WBC count >50 $x10^9/L$); HR: all non-SR cases. HR patients were assigned to undergo allogeneic SCT. Provided sufficient CD34+ cells were previously collected ($>2x10^6/kg$) upon recovery from high doses cytarabine, SR patients and HR patients excluded from allo-SCT and aged 65 years or less were randomized between autologous SCT and high doses consolidation therapy (R2). HR/SR patients unable to be randomized in R2 because of inadequate blood stem cell yield received intermediate-dose consolidation. Patients randomized to experimental arm were excluded from outcome analysis.

Protocol-3: since May 2014 to April 2017, patients received induction according to Ida-FLA course, (Cytarabine 2000 mg/sqm on days 1-4; Fludarabine 30 mg/sqm on days 1-4; Idarubicin 10 mg/sqm on days 2-4). High-dose Cytarabine (3000 mg/sqm bid days 1, 3, 5) was used as first consolidation in patients aged < 61 years attaining complete remission (CR). Patients with persistent disease (*i.e.* > 5% BM blasts at hematopoietic recovery) after first course received a salvage regimen (Clofarabine-based). In post CR phase, patients were stratified according to European Leukemia Net 2010 guidelines [2]. Patients in adverse-risk category were allocated to allogeneic HSCT from matched related or unrelated donor. Patients in intermediate category were allocated to allogeneic SCT if a related donor was available. Patients in favorable-risk ELN category and high-risk disease not eligible to allogeneic SCT received up to two additional courses with high dose cytarabine.

S2. Assessment of Multi-Lineage Dysplasia by Multiparameter Flow Cytometry

A minimum of 250,000 cells per tube was acquired. Instrument setup, calibration and quality control were performed in order to assure measures' stability [3]. For data analysis, Infinicyt (Cytognos SL, Salamanca, Spain) software was used. Some major BM cell compartments were

identified on the basis of forward (FSC) and sideward (SSC) light scatter characteristics and their reactivity for CD45 as in the Figure below:

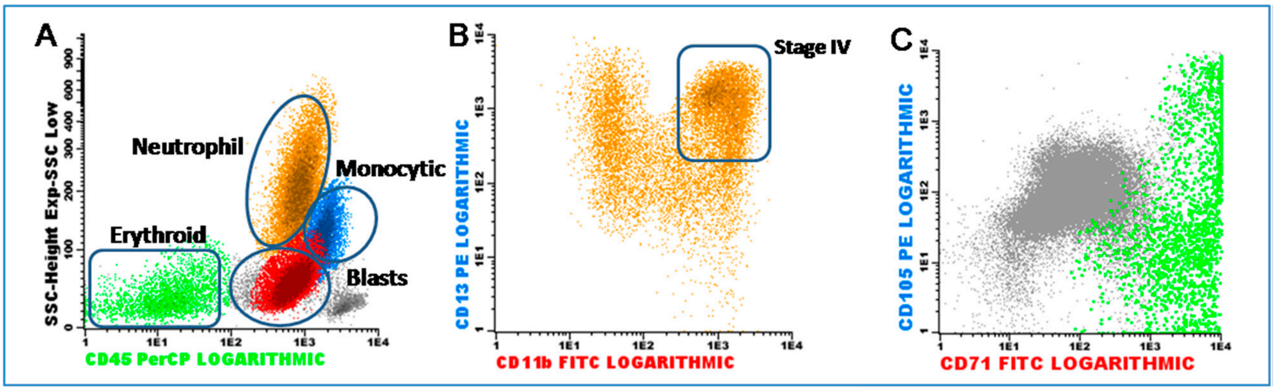

These subsets were: (i) blasts; (ii) maturing neutrophil compartment; (iii) mature monocytic compartment; (iv) mature erythroid compartment. In order to estimate dysplasia, we considered neutrophil and erythroid compartments only; monocytic compartment was excluded from IPS because often clonally involved in AML and thus inadequate to assess MLD. As for neutrophil compartment, it was identified on the basis of CD45 dim expression with high SSC signal. Consistently with WHO criteria for morphological dysplasia, the analysis was then focused on the more mature stage, by gating cells co-expressing CD13 and CD11b or expressing CD16. The expression of CD65 and cyMPO were evaluated on whole granulocytic compartment. As for erythroid compartment, it was selected by negativity for CD45 and CD34 with low SSC signal. With respect to MDS-related approach [4]. CD235a was substituted by CD105. Dysplasia was appraised for neutrophil and erythroid compartments through an immuno-phenotypic score (IPS) including 17 parameters (13 for neutrophil and 4 for erythroid compartment) as in the Table below:

**Phenotypic parameter**
**Neutrophil compartment**
FSC MI
SSC MI
CD45 MFI
CD11b MFI
CD13 MFI
CD33 MFI
CD15 MFI
CD64 MFI
CD65 MFI
MPO MFI
CD16hi %
CD56 %
CD14 %
**Erythroid compartment**
CD36 MFI
CD36 %
CD71 MFI
CD105 MFI

Cell compartments were considered not assessable for dysplasia when not detectable as at least 0.01% of total BM cells. Parameters included by IPS were expressed as percentage of positive cells for an antigen within a cell compartment and/or its mean fluorescence intensity (MFI; arbitrary relative linear units, scaled from 0 to 10^4, normalized upon a control). BM samples from healthy donors were

used to define normal phenotypic profile as the interval between mean value ± two standard deviations (SD) for each parameter. A score was calculated for each parameter: 0.5, 1 or 2 was assigned when the value was between the mean ± 2 SD and the mean ± 3 SD, between the mean ± 3 SD and the mean ± 4 SD and, over the mean ± 4 SD of the normal profile, respectively.

Supplemental Figures and Legends

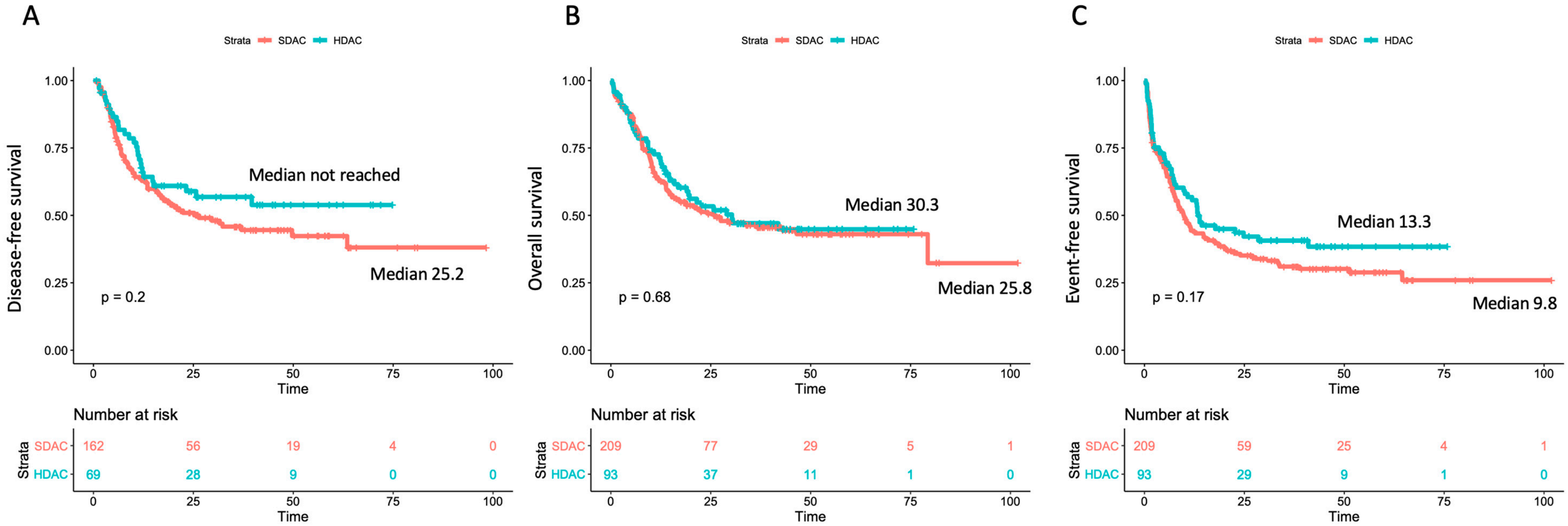

Figure S1. Disease-free (**A**), overall (**B**), and event-free (**C**) survival according to the induction regimen, either including standard dose cytarabine (SDAC) or high dose cytarabine (HDAC).

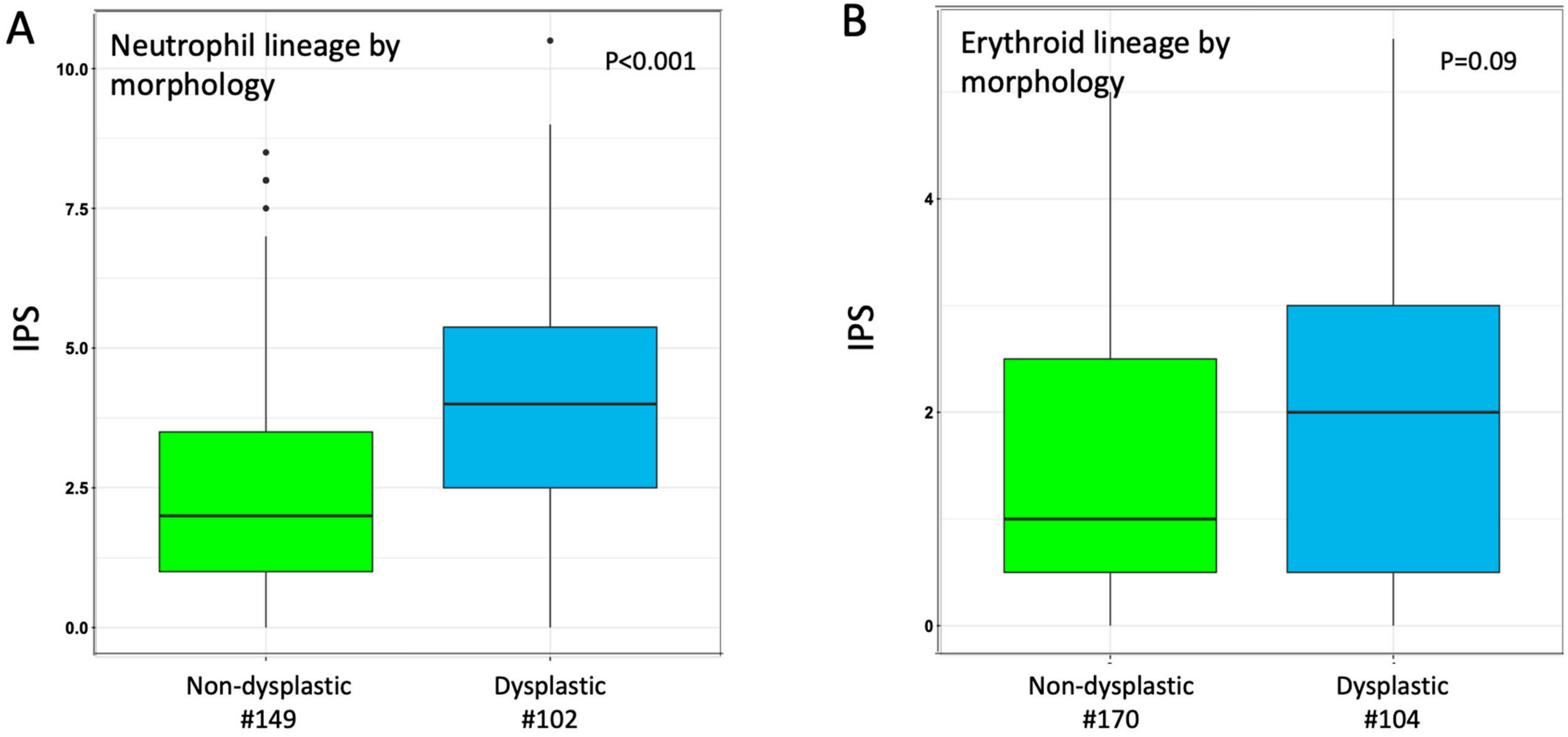

Figure S2. Distribution of phenotypic scores in single cell lineage, either neutrophil (**A**) or erythroid (**B**), according to dysplasia by morphology. Fifty-one and twenty-eight patients were not assessable for dysplasia by morphology in neutrophil and erythroid cell lineage, respectively.

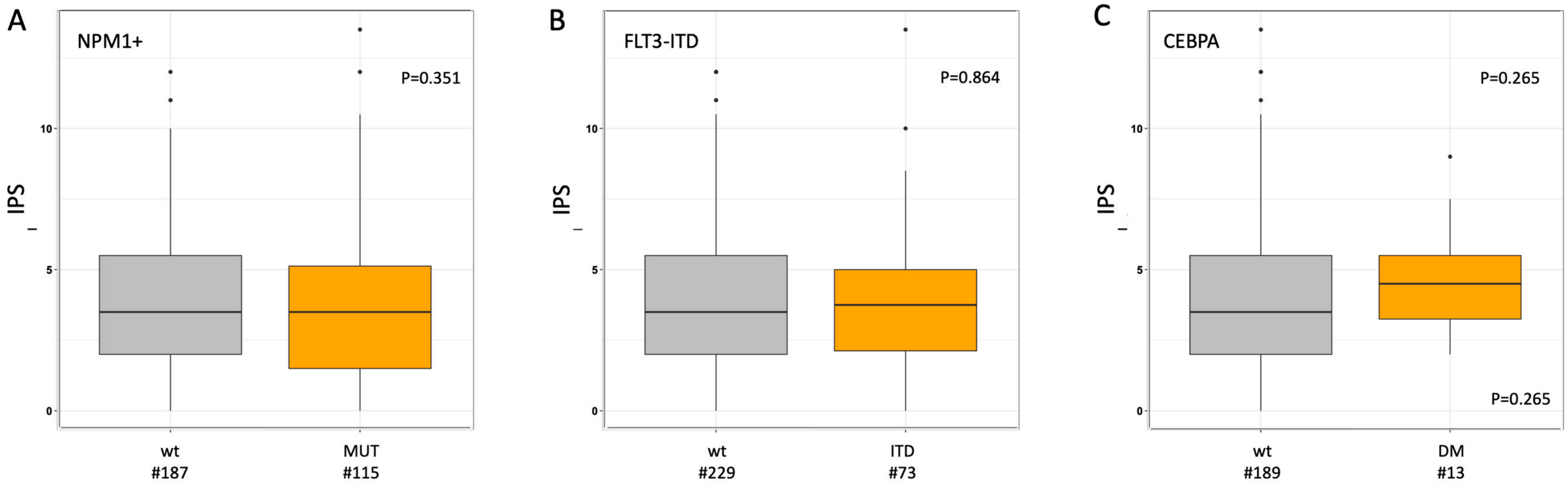

Figure S3. Distribution of IPS values according to genotype for NPM1 status (**A**), presence of FLT3-ITD (**B**), CEBPA status (**C**).

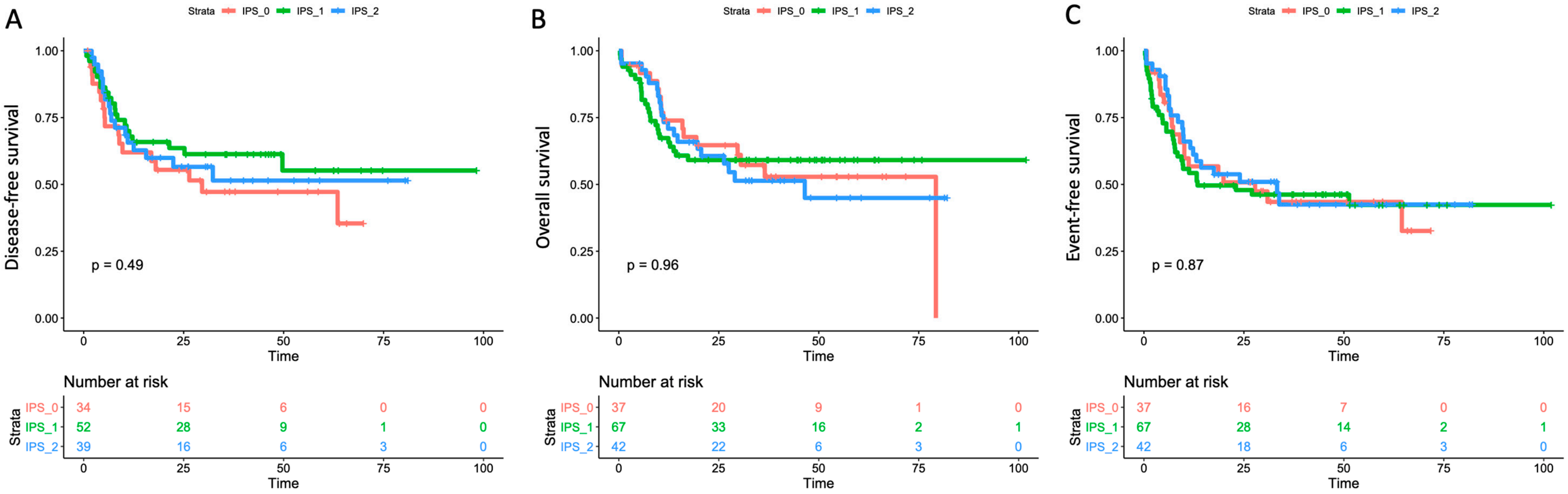

Figure S4. Disease-free (**A**), overall (**B**), and event-free (**C**) survival according to number of dysplastic lineages by immunophenotypic score (IPS) in WHO subset of AML with recurrent genetic abnormalities (RGA).

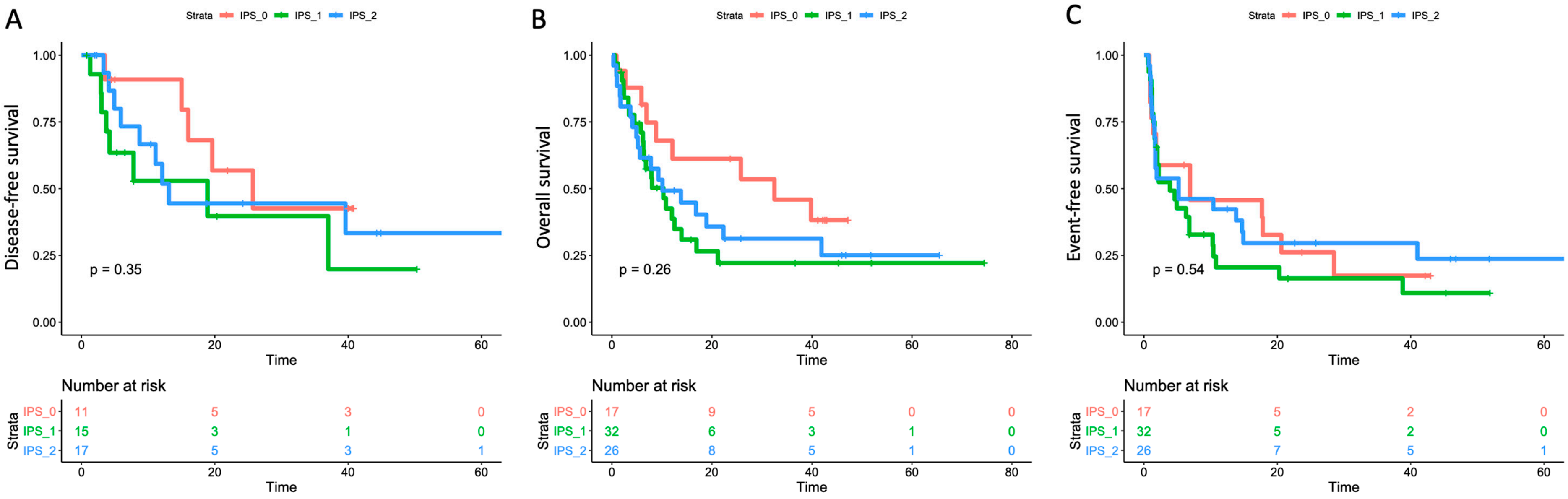

Figure S5. Disease-free (**A**), overall (**B**), and event-free (**C**) survival according to number of dysplastic lineages by immunophenotypic score (IPS) in WHO subset of AML with myelodysplasia-related changes (MRC).

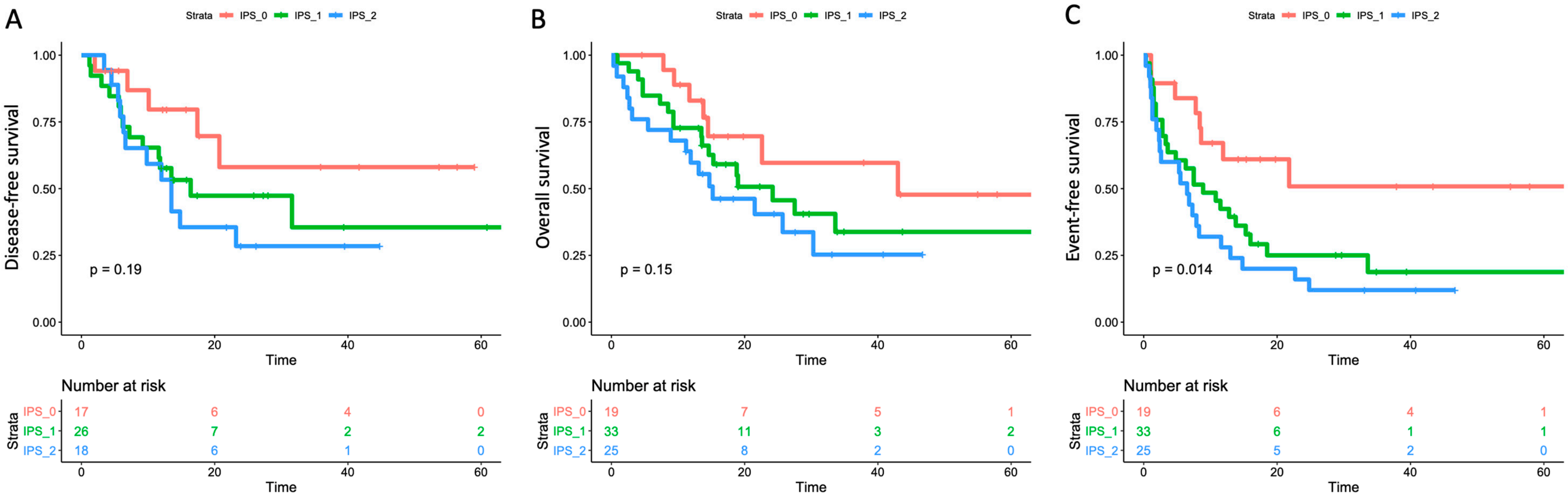

Figure S6. Disease-free (**A**), overall (**B**), and event-free (**C**) survival according to number of dysplastic lineages by immunophenotypic score (IPS) in WHO subset of AML not-otherwise specified (NOS).

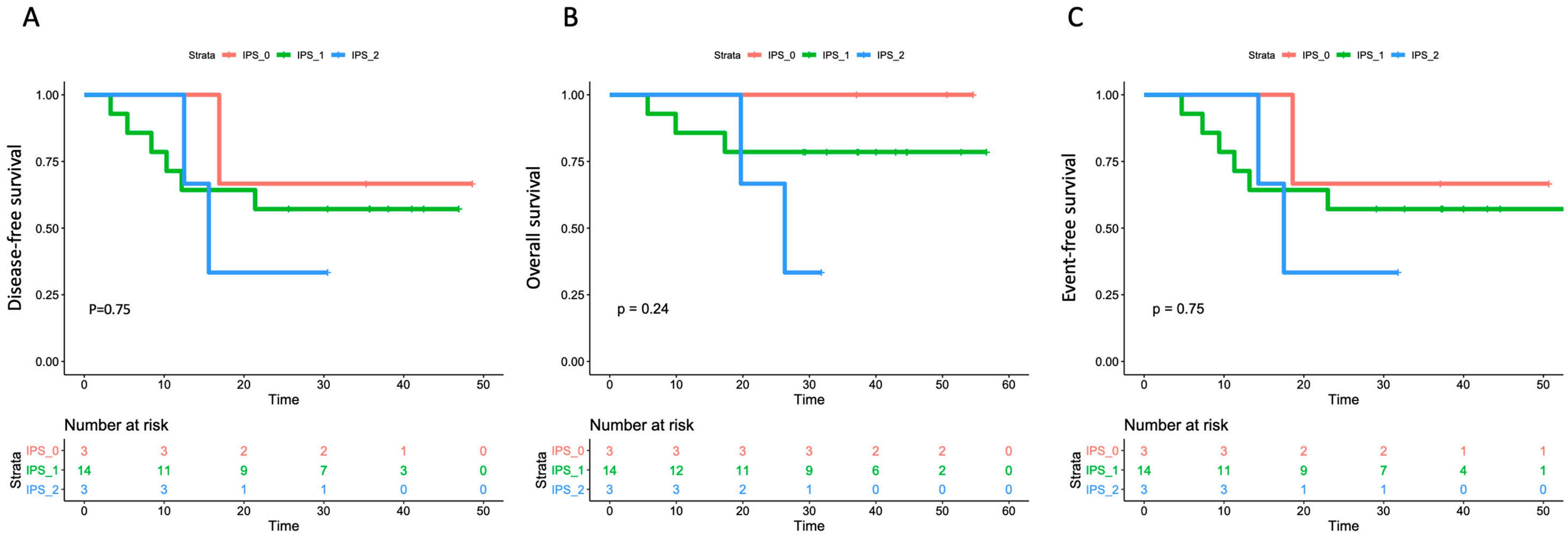

Figure S7. Disease-free (**A**), overall (**B**), and event-free (**C**) survival according to number of dysplastic lineages by immunophenotypic score (IPS) in favorable-risk karyotype.

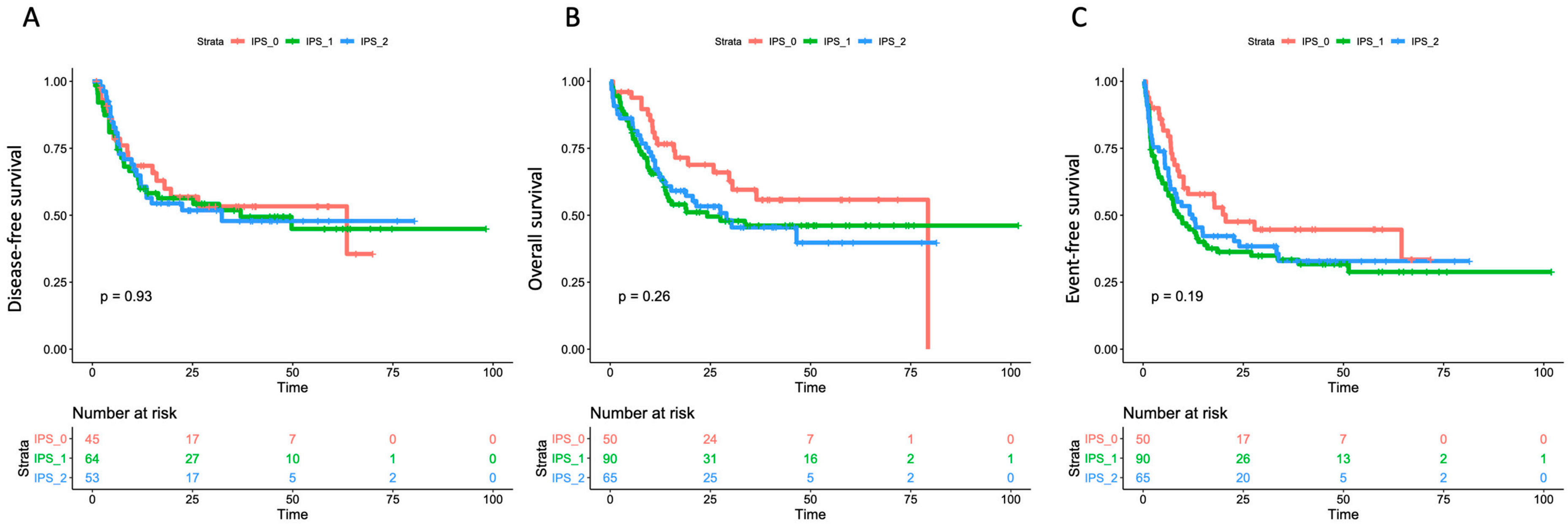

Figure S8. Disease-free (**A**), overall (**B**), and event-free (**C**) survival according to number of dysplastic lineages by immunophenotypic score (IPS) in intermediate-risk karyotype.

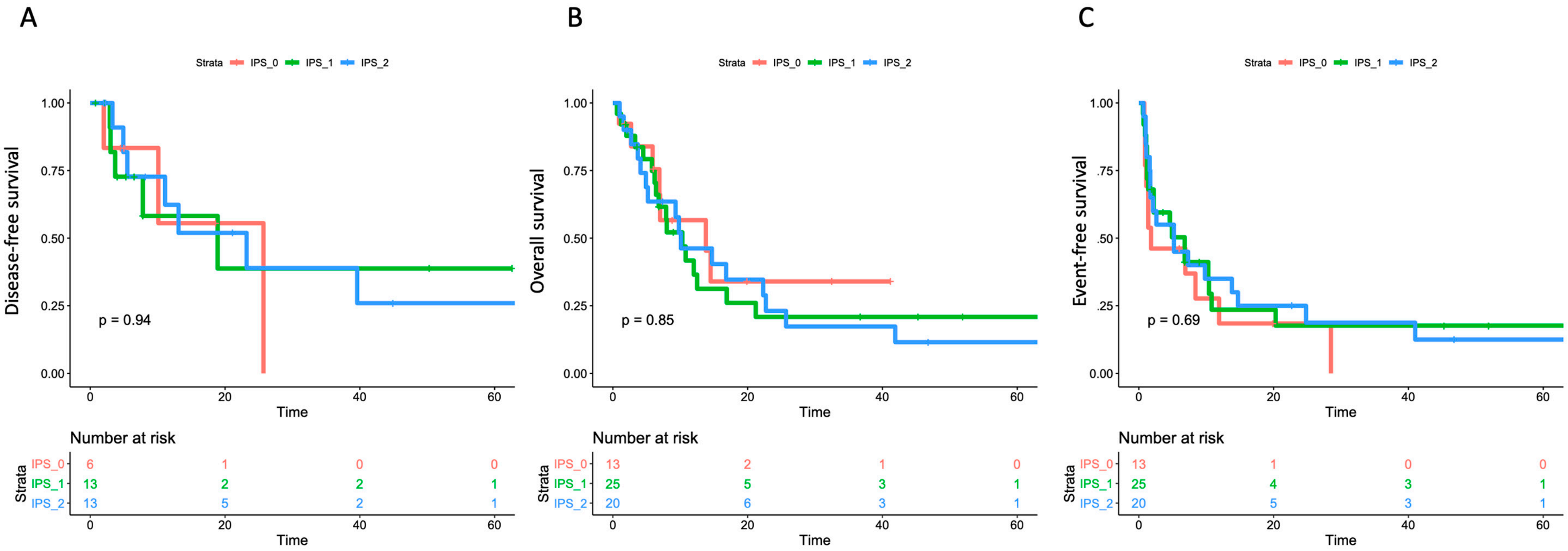

Figure S9. Disease-free (**A**), overall (**B**), and event-free (**C**) survival according to number of dysplastic lineages by immunophenotypic score (IPS) in adverse-risk karyotype.

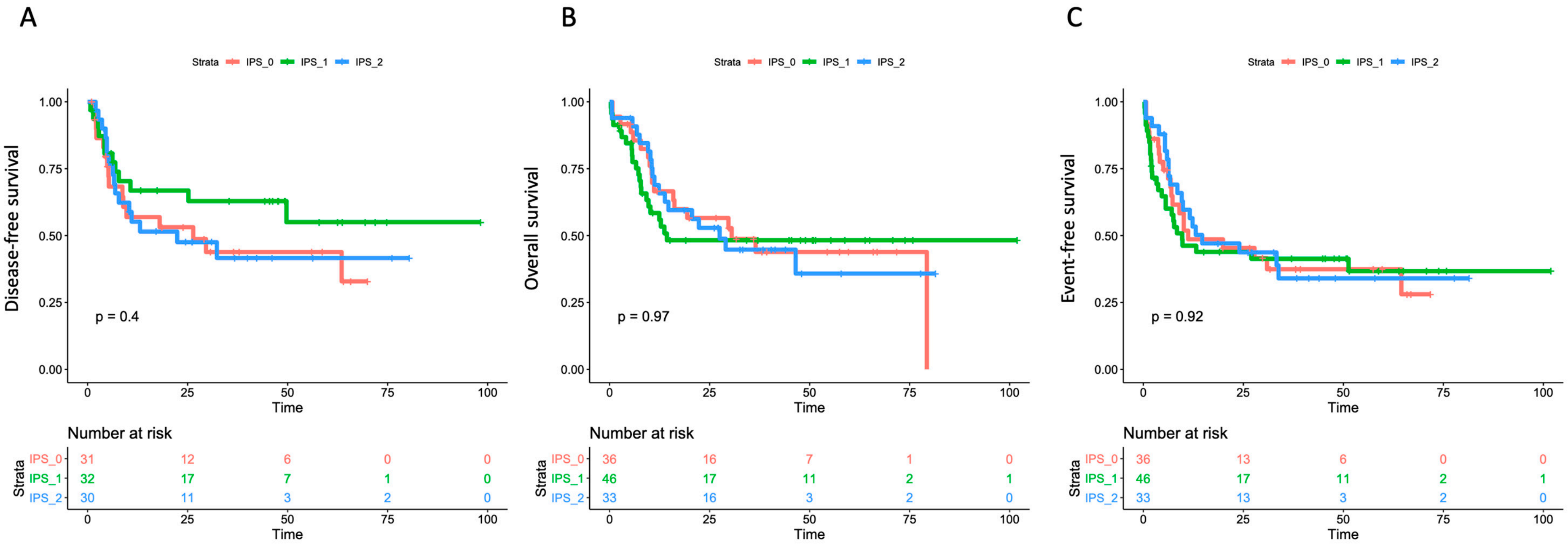

Figure S10. Disease-free (**A**), overall (**B**), and event-free (**C**) survival according to number of dysplastic lineages by immunophenotypic score (IPS) in NPM1-mutated patients.

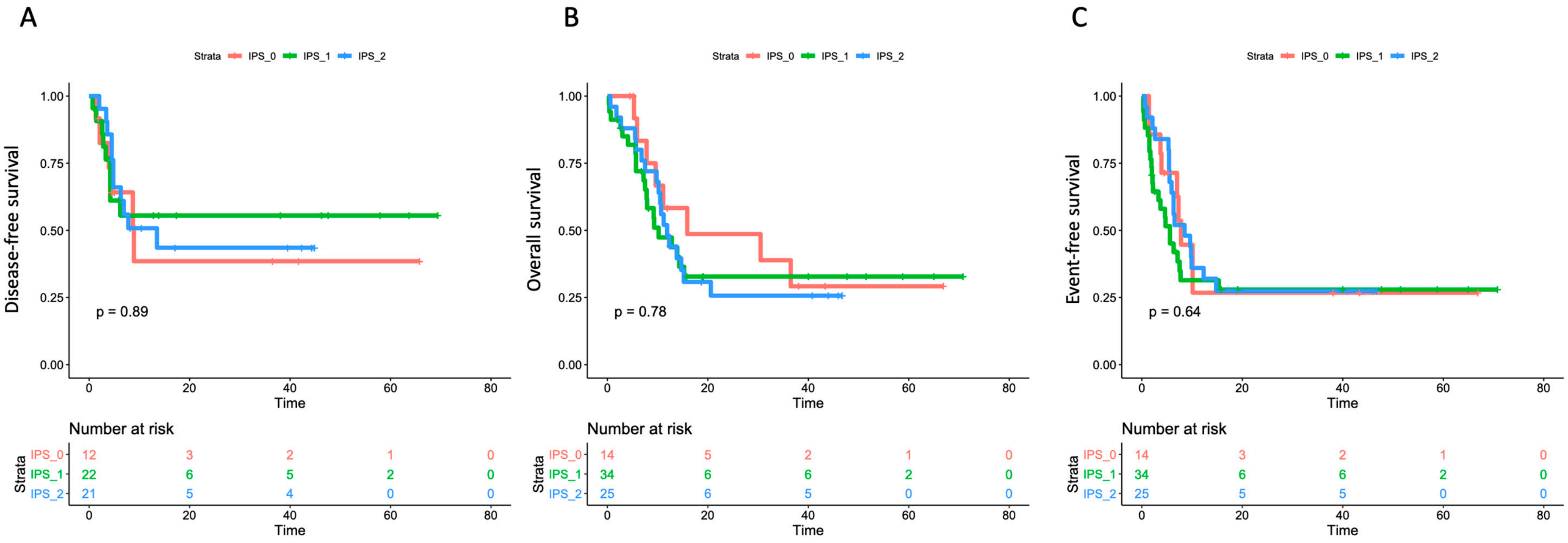

Figure S11. Disease-free (**A**), overall (**B**), and event-free (**C**) survival according to number of dysplastic lineages by immunophenotypic score (IPS) in FLT3-ITD patients.

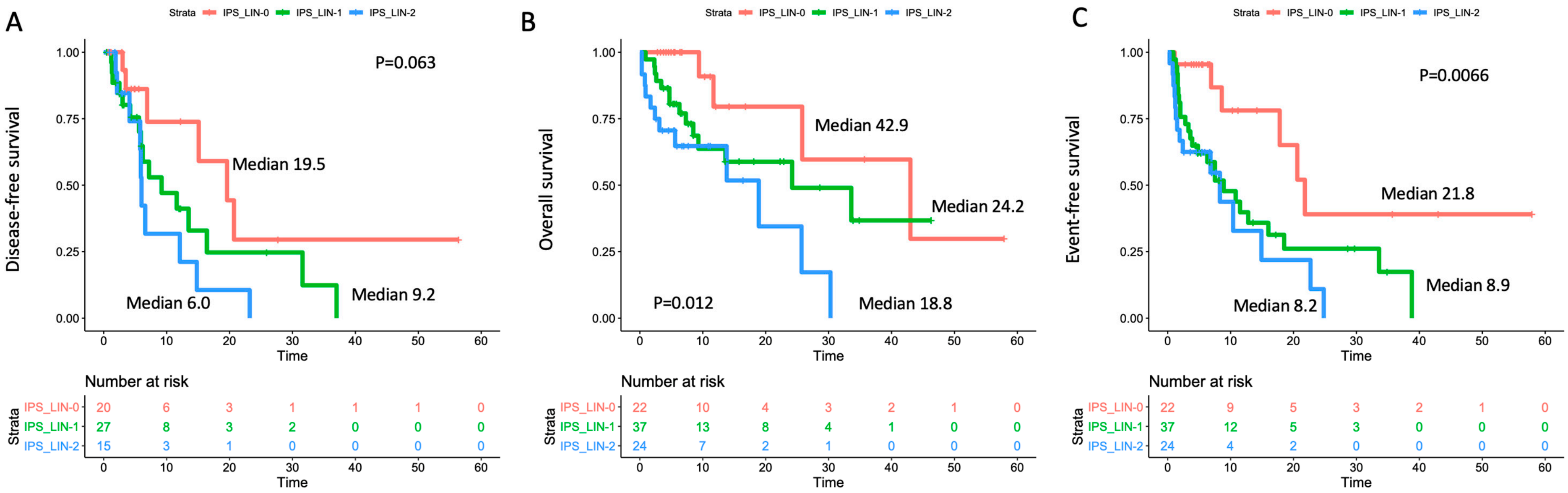

Figure S12. Disease-free (**A**), overall (**B**), and event-free (**C**) survival according to number of dysplastic lineages by immunophenotypic score (IPS) in triple-negative AML with censoring at allogeneic transplant.

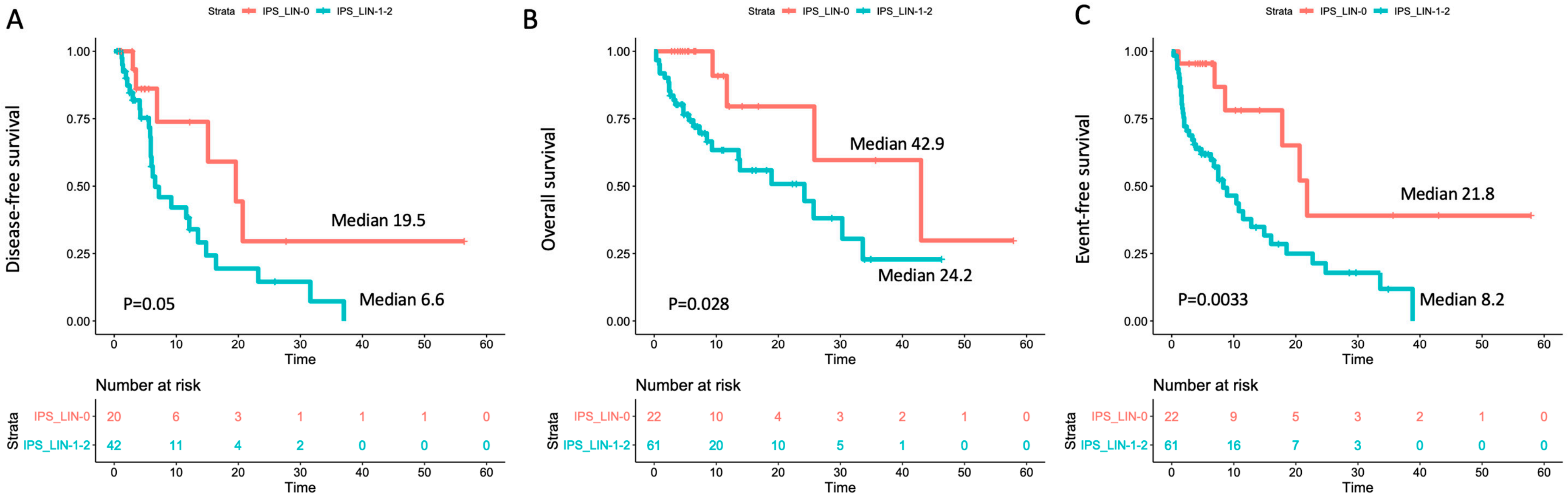

Figure 13. Disease-free (**A**), overall (**B**), and event-free (**C**) survival according to IPS in triple-negative AML with censoring at allogeneic transplant. Patients with no dysplastic lineage (IPS_0) are compared to patients with 1 or 2 dysplastic lineages (IPS_1-2).

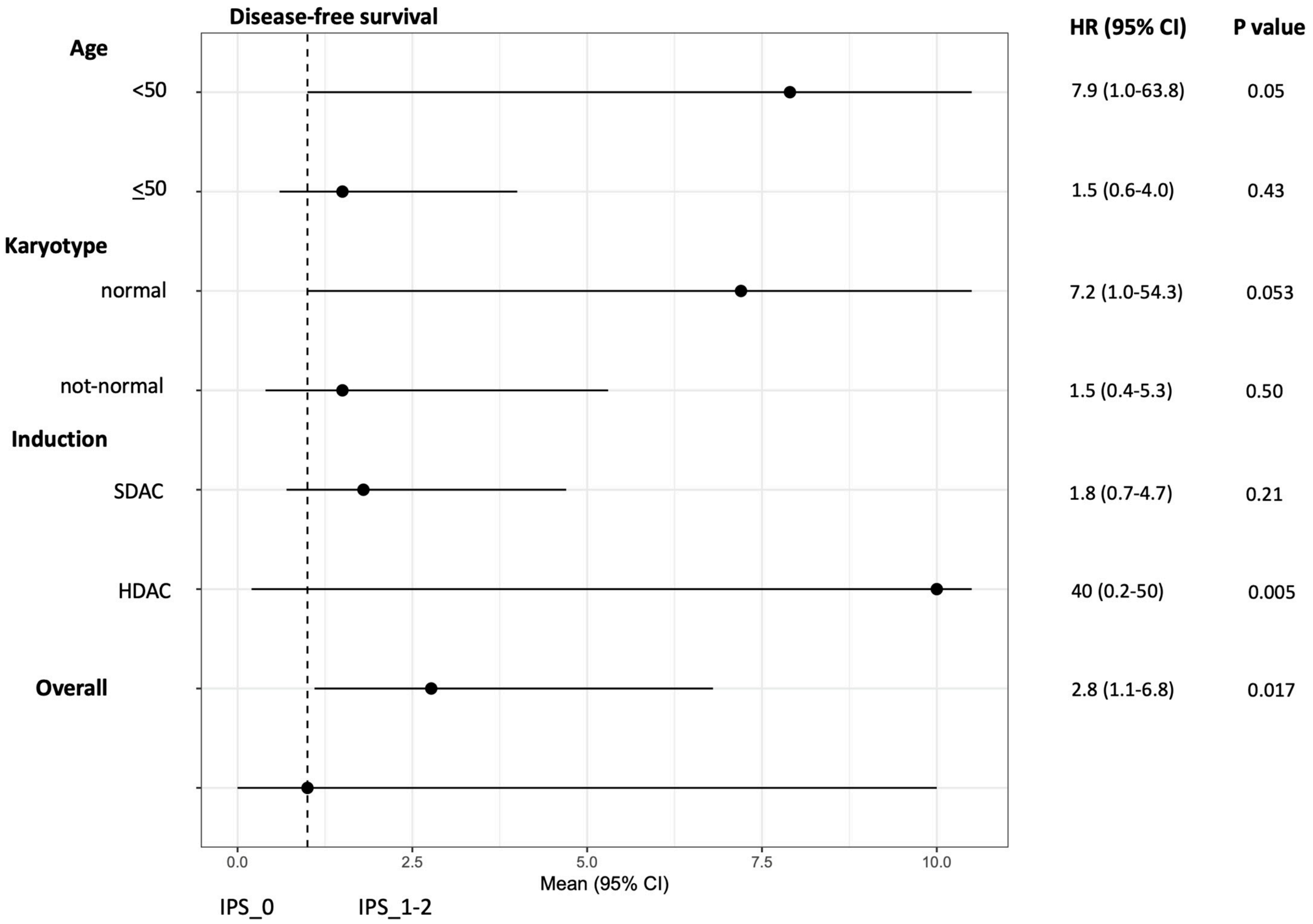

Figure S14. Effect of IPS on DFS. Forest plot depicting the effect of IPS-related group IPS_0 versus IPS_1-2) on DFS in triple-negative AML according to main patient and disease characteristics. HR, hazard ratio; 95% CI, confidence interval; SDAC: standard-dose cytarabine; HDAC: high-dose cytarabine.

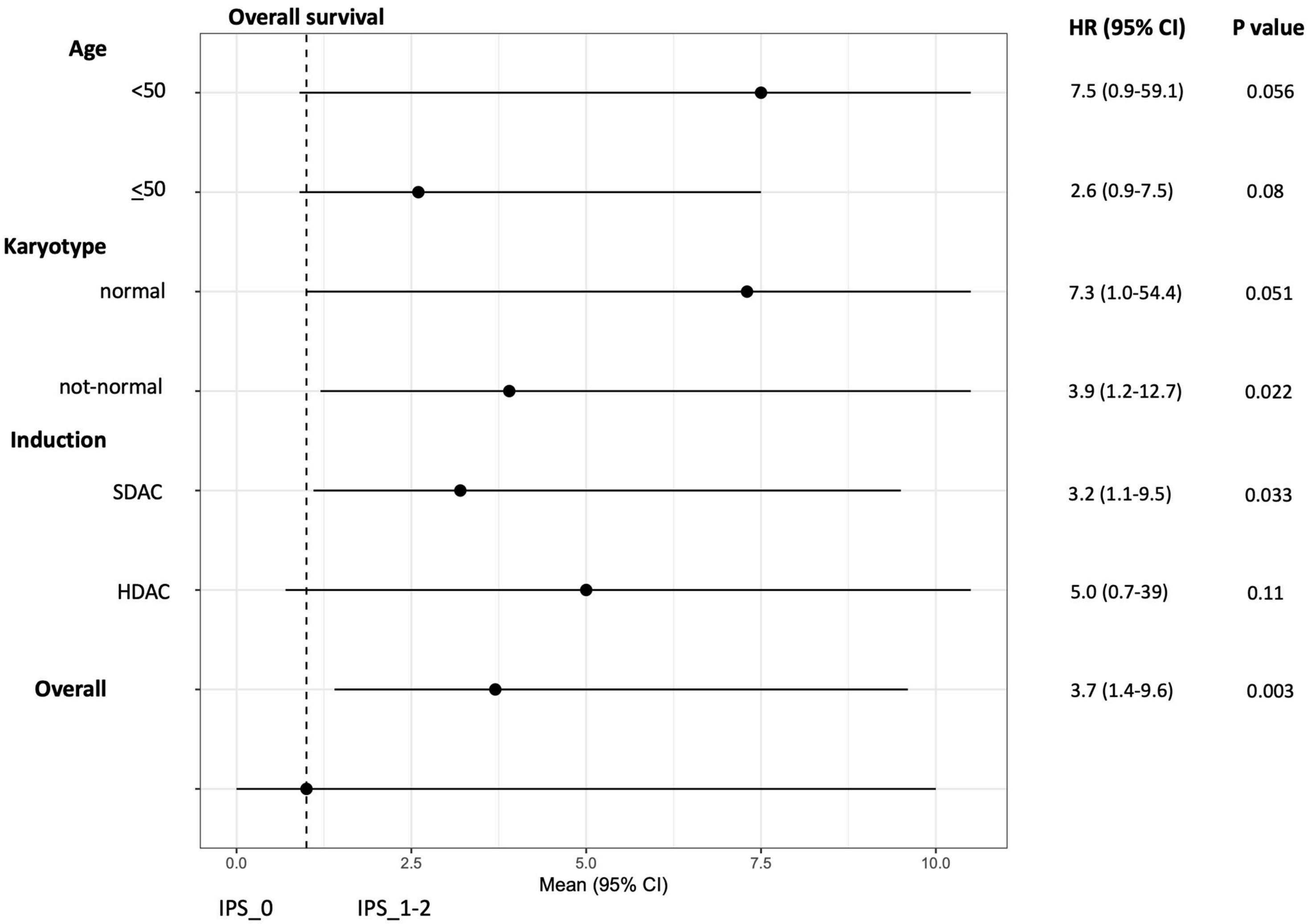

Figure S15. Effect of IPS on OS. Forest plot depicting the effect of IPS-related group IPS_0 versus IPS_1-2) on OS in triple-negative AML according to main patient and disease characteristics. HR, hazard ratio; 95% CI, confidence interval; SDAC: standard-dose cytarabine; HDAC: high-dose cytarabine.

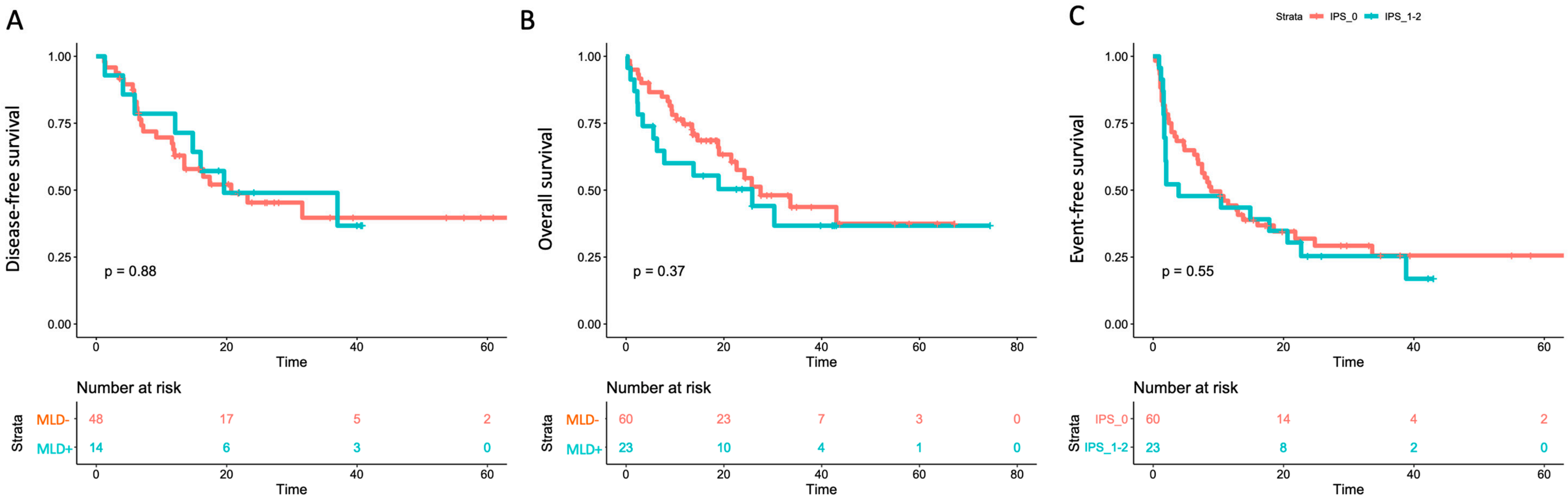

Figure S16. Outcome according multi-lineage dysplasia as assessed by morphology in triple-negative AML.

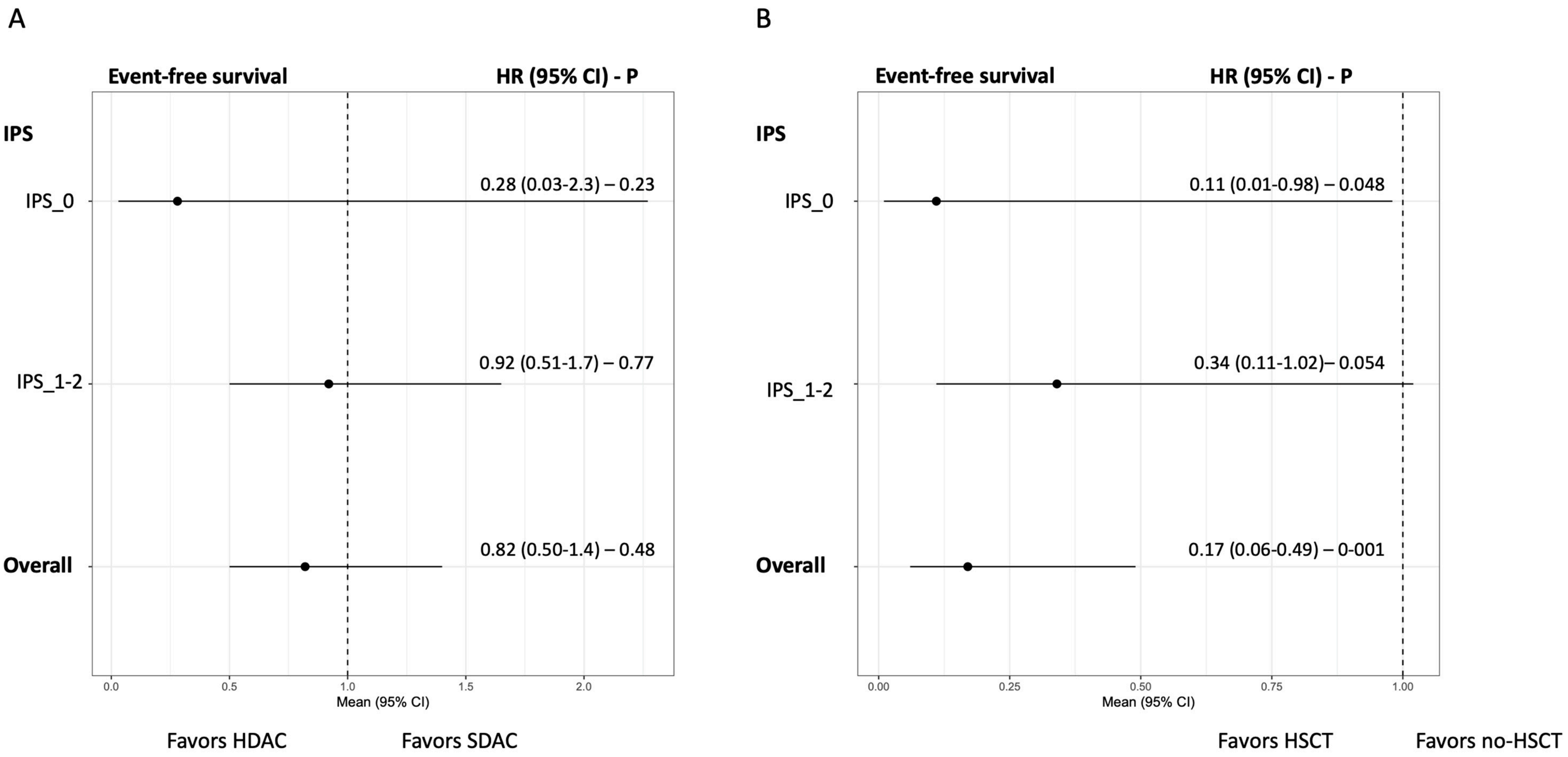

Figure S17. Effect of treatment-related covariates on EFS. Forest plot depicting the effect of induction intensity (**A**) and allogeneic transplant [(**B**), as time-dependent covariate] in IPS-related group (IPS_0 versus IPS_1-2) on EFS. HR, hazard ratio; 95% CI, confidence interval; SDAC: standard-dose cytarabine; HDAC: high-dose cytarabine; HSCT: allogeneic transplant.

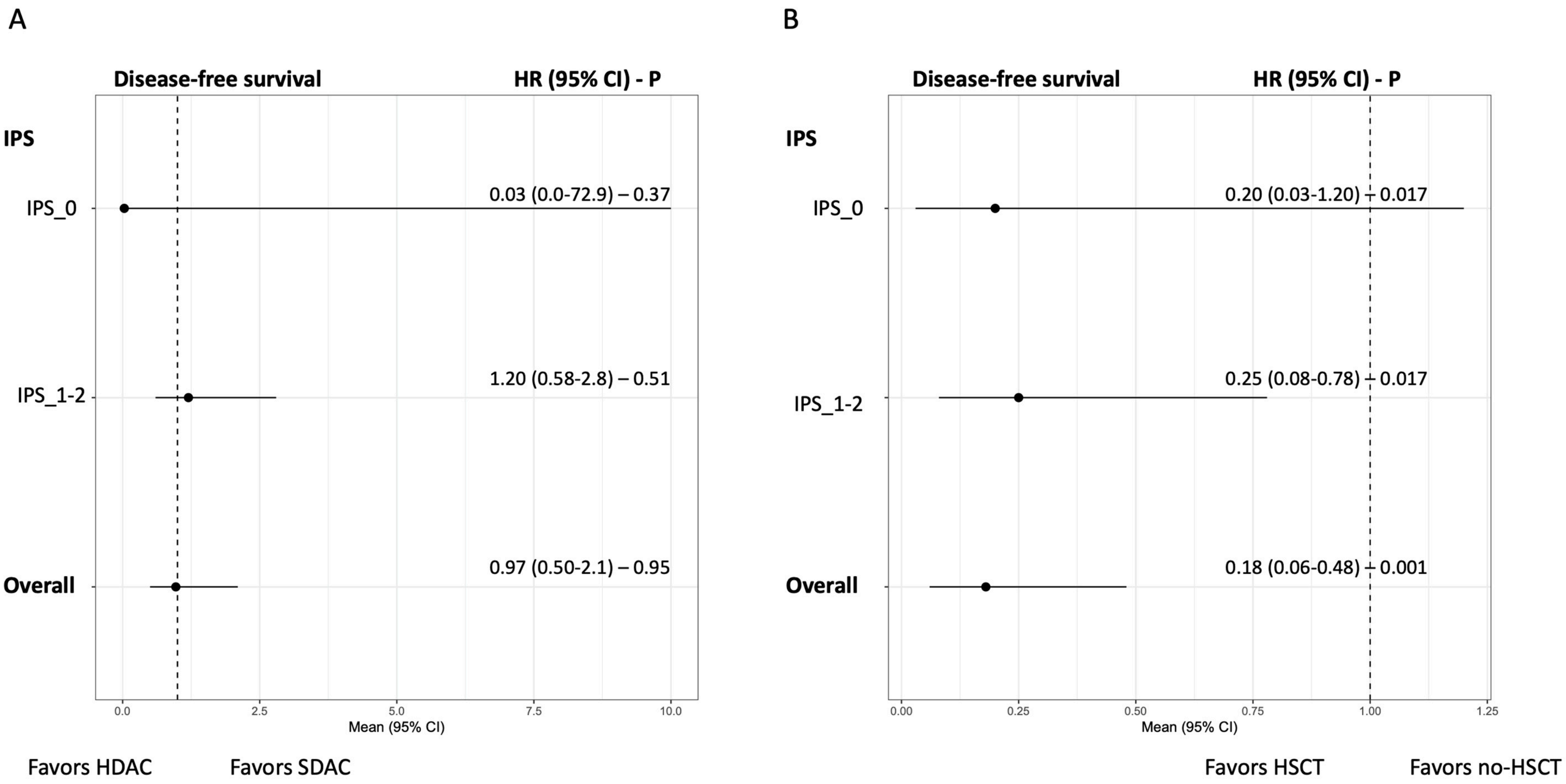

Figure S18. Effect of treatment-related covariates on DFS. Forest plot depicting the effect of induction intensity (A) and allogeneic transplant [(B), as time-dependent covariate] in IPS-related group (IPS_0 versus IPS_1-2) on DFS. HR, hazard ratio; 95% CI, confidence interval; SDAC: standard-dose cytarabine; HDAC: high-dose cytarabine; HSCT: allogeneic transplant.

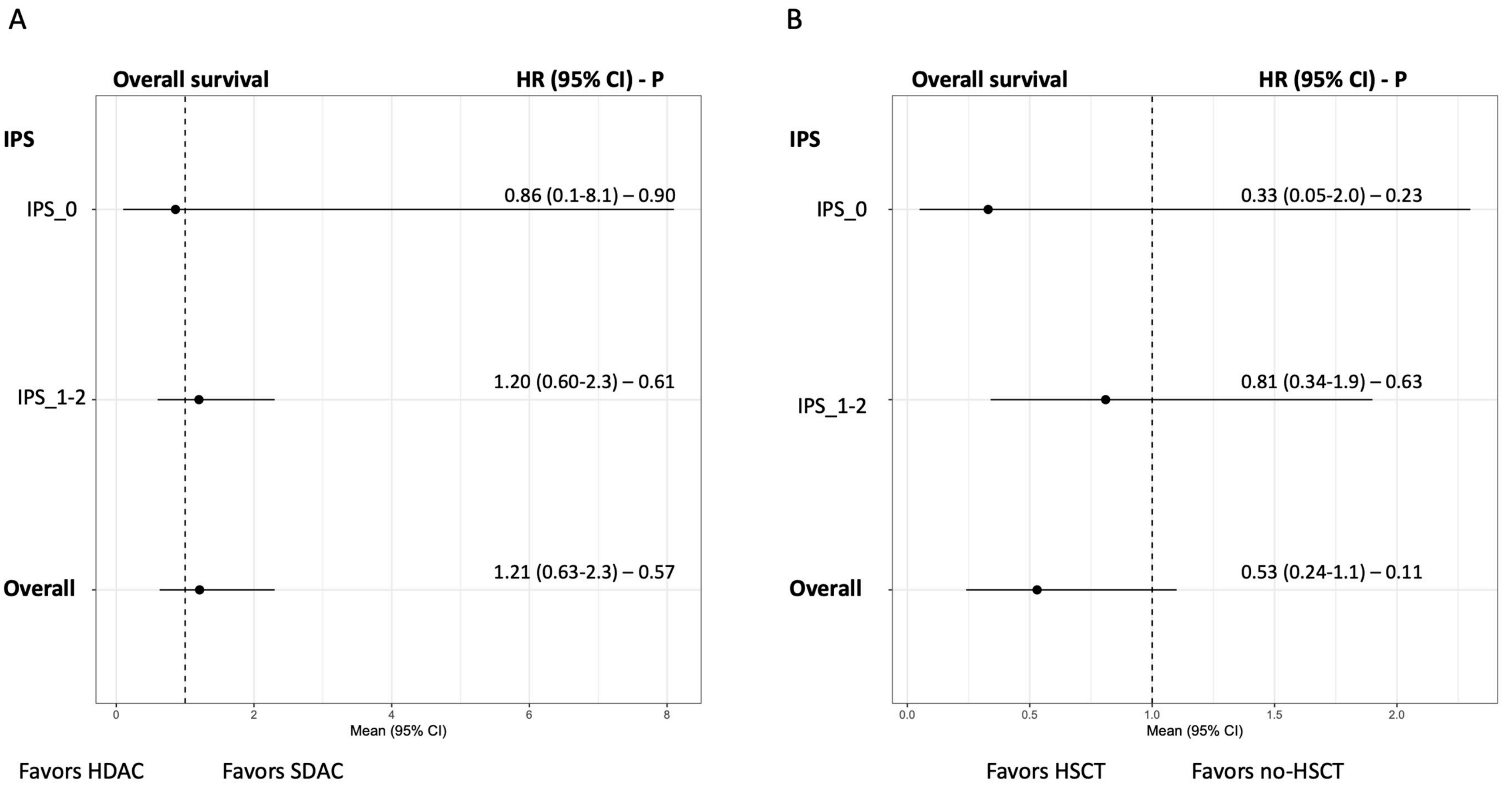

Figure S19. Effect of treatment-related covariates on OS. Forest plot depicting the effect of induction intensity (A) and allogeneic transplant [(B) as time-dependent covariate] in IPS-related group (IPS_0 versus IPS_1-2) on OS. HR, hazard ratio; 95% CI, confidence interval; SDAC: standard-dose cytarabine; HDAC: high-dose cytarabine; HSCT: allogeneic transplant.

Supplemental Tables and Results

Table S1. Characteristics of patients according to treatment protocol, separated according to induction regimen including either standard (SDAC) or high dose (HDAC) cytarabine.

	**SDAC** **n = 209 (69.2%)**	**HDAC** **n = 93 (30.8%)**	***P* value**
**Age,** median (range)	56 (18-75)	54 (20-75)	0.317
**WBC,** x10⁹/L, median (range)	19.2 (0.6–435.0)	8.6 (1.1-289)	0.121
**Hb,** g/dL, median (range)	9.1 (3.9–14.5)	8.9 (3.4-14.9)	0.372
**Plt,** x10⁹/L, median (range)	52 (5–373)	46 (3-272)	0.580
**Bone marrow blasts,** %, median (range)	90 (20–100)	90 (20-100)	0.653
**Karyotype,** n (%)			
Favorable	15 (7.2)	5 (5.4)	0.954
Normal	128 (61.2)	46 (49.4)	
Intermediate, non-normal	14 (6.7)	17 (18.3)	
Adverse	36 (17.2)	22 (23.7)	
Lack of growth	16 (7.7)	3 (3.2)	
**Molecular genetics,** n (%)			
NPM1-mutated	86 (41.1)	29 (31.2)	0.123
FLT3-ITD	55 (26.3)	18 (19.4)	0.244
FLT3-TKD	4 (1.9)	3 (3.2)	0.680
CEBPA-DM	7 (3.3)	6 (6.4)	0.230
**ELN 2010 risk groups,** n (%)			
Favorable	65 (31.1)	25 (26.9)	0.217
Intermediate	108 (51.7)	46 (49.5)	
Adverse	36 (17.3)	22 (23.7)	
**Induction regimen,** n (%)			
3+7	50 (23.9)	-	-
ICE	159 (76.1)	-	-
HDS	-	28 (30.1)	-
Ida-FLA	-	65 (69.9)	-
**CR rate,** n (%)			

After course 1	125 (59.8%)	57 (61.3%)	0.898
After course 2	162 (77.5%)	69 (74.2%)	0.558

Table S2. Characteristics of patients according to number of dysplastic lineages by immunophenotypic score (IPS).

	**IPS_0** **n = 74 (24.5%)**	**IPS_1** **n = 133 (44.0%)**	**IPS_2** **n = 95 (31.5%)**	***P* value**
**Age,** median (range)	57 (23-75)	55 (18–75)	55 (20-75)	0.384
**WBC,** x10⁹/L, median (range)	10.0 (0.6–435.0)	19.4 (0.8–415)	13.7 (1.0-289)	0.336
**Hb,** g/dL, median (range)	9.3 (4.2–14.5)	9.0 (3.4–14.2)	8.9 (3.9-14.9)	0.115
**Plt,** x10⁹/L, median (range)	57 (7–321)	51 (3–373)	40 (10-272)	**0.026**
**Bone marrow blasts,** %, median (range)	90 (20–100)	90 (20–100)	80 (20-100)	0.378
**Karyotype,** n (%)				
Favorable	3 (4.0)	14 (10.5)	3 (3.2)	0.061
Normal	38 (51.4)	78 (58.7)	58 (61.0)	
Intermediate, non-normal	12 (16.2)	12 (9.0)	7 (7.4)	
Adverse	13 (17.6)	25 (18.8)	20 (21.0)	
Lack of growth	8 (10.8)	4 (3.0)	7 (7.4)	
**Molecular genetics,** n (%)				
NPM1-mutated	36 (48.6)	46 (34.6)	33 (34.7)	0.098
FLT3-ITD	14 (18.9)	34 (25.6)	25 (26.3)	0.285
FLT3-TKD	2 (2.7)	2 (1.5)	3 (3.2)	0.792
CEBPA-DM	1 (1.4)	6 (4.5)	6 (6.4)	
**WHO Classification,** n (%)				
Recurrent genetic abnormalities	37 (50.0)	67 (50.3)	42 (44.2)	0.946
Therapy-related	1 (1.4)	1 (0.8)	2 (2.1)	
Myelodisplasia-related changes	17 (23.0)	32 (24.1)	26 (27.4)	
Not otherwise classified	19 (25.6)	33 (24.8)	25 (26.3)	
**ELN 2010 risk groups,** n (%)				
Favorable	23 (31.1)	39 (29.3)	28 (29.5)	0.862
Intermediate-1	29 (39.2)	60 (45.1)	40 (42.1)	
Intermediate-2	9 (12.1)	9 (6.8)	7 (7.4)	

Adverse	13 (17.6)	25 (18.8)	20 (21.0)

Differences between treatment groups were evaluated using Kruskal-Wallis test for continuous variables and Fisher exact tests or χ2 for categorical variables. Values in bold are statistically significant ($P < 0.05$). Abbreviations: WBC, white blood cells; Hb, hemoglobin, Plt, platelets; ELN, European Leukemia Net.

References of Methods' Section

1. Bassan R, Intermesoli T, Masciulli A, et al. Randomized trial comparing standard vs sequential high-dose chemotherapy for inducing early CR in adult AML. Blood Advances. 2019;3(7):1103–1117.
2. Döhner H, Estey EH, Amadori S, et al. Diagnosis and management of acute myeloid leukemia in adults: recommendations from an international expert panel, on behalf of the European LeukemiaNet. Blood. 2010;115(3):453–474
3. Owens MA, Vall HG, Hurley AA, Wormsley SB. Validation and quality control of immunophenotyping in clinical flow cytometry. Journal of Immunological Methods. 2000;243(1–2):33–50.
4. Matarraz S, López A, Barrena S, et al. (2010) Bone marrow cells from myelodysplastic syndromes show altered immunophenotypic profiles that may contribute to the diagnosis and prognostic stratification of the disease: a pilot study on a series of 56 patients. Cytometry. Part B, Clinical cytometry, 78, 154–68.
